# Observations on Permanent Strictures of the Urethra

**Published:** 1833-10

**Authors:** Fredk. Tyrrell

**Affiliations:** New Bridge street


*To the Editor of the Medical Quarterly Review.*

Sir: Should you consider the accompanying observations upon permanent stricture of the urethra to be worthy the attention of the profession, I shall feel obliged by your giving them a place in the Medical Quarterly. My object in offering them is to direct the attention of the junior members to this important subject, and I shall be highly gratified if the perusal of the paper should deter any of them from adopting a practice, which, though occasionally attended with eclat, is, according to my own experience, fraught with the greatest danger. I intend, at an early opportunity, to forward to you the results of my observations upon the subjects of inflammatory and spasmodic stricture, should you have space in a future number for their insertion.

I have the honour to remain yours respectfully,

Fredk. Tyrrell.

*New Bridge street; September 18th,* 1833.

*Observations on Permanent Strictures of the Urethra.*

The symptoms of permanent stricture are, 1st, a more frequent desire than usual to void the urine, with some slight irregularity in the stream, not noticed perhaps by the patient, and some little dribbling from the passage after he thinks he has emptied the bladder. 2dly. After a time the alteration in the stream becomes so great as to attract the patient's attention; it is usually diminished in size, is twisted, as a screw, as it quits the meatus, or it spreads or divides, sometimes into two, sometimes into numerous smaller streams; occasionally it quits the meatus at an angle, being projected upwards, downwards, or laterally. The patient occasionally complains of uneasiness or pain about the perinæum. 3dly. As the disease progresses, the stream becomes further diminished, and at length the urine merely passes *guttatim.* The urgency for micturition is greatly increased, so that the patient is scarcely a quarter of an hour without the inclination to pass his urine, and then discharges but a small quantity, and that with great effort. The violent straining made use of by some patients frequently induces hernia, hæmorrhoids, or prolapsus ani. The bladder, in the aggravated state of the disease, is seldom completely emptied, and the urine remaining in it undergoes some change, which creates disease of the mucous membrane of the organ. The muscular coat is sometimes enormously thickened, from its almost constant and violent exertion to expel the urine. Now and then sacculi are formed, from the mucous membrane protruding between the fibres of the muscular coat. Usually, years elapse from the occurrence of the first symptoms before the disease reaches this distressing condition; but occasionally it arrives at this extent in the course of a few months.

The *causes* of permanent stricture are principally chronic inflammation of the mucous membrane of the urethra, subsequent to gonorrhœa; inflammation, the result of violence to the mucous membrane, as in the introduction of a bougie, sound, or catheter; or from external injury, as a blow upon the part.

The *proximate cause*, upon careful examination, appears to be a partial thickening of the mucous membrane, from deposition of fibrine, which is thrown out in the substance of the membrane, and in the subjacent cellular tissue. The extent of this deposit varies extremely, and is seldom equal around the canal, being most frequently greater at the inferior or lateral parts than at the superior. Sometimes it produces an elevation of the membrane of scarcely an eighth of an inch in extent, whilst at other times the thickening extends for the space of an inch or more. The condition of the diseased portion of the canal is generally firm like callus, and more resisting than the natural structure; but sometimes it is soft, and easily broken down: in the first instance its vascularity is trifling; in the latter, it is very great. If the calibre of the canal be diminished by a deposit equally around it, the stream of urine is simply diminished in size; but, when the deposit is unequal, the stream either spreads, divides, or scatters, as it quits the meatus.

*Situation of Permanent Stricture.* From very extensive experience and careful observation, I am satisfied that permanent stricture occurs most frequently (fully in the proportion of twenty to one,) in that portion of the urethra which is surrounded by the accelerator urinæ muscle. The length of the canal itself, when the penis is moderately stretched, will be found, on the average, to be about eight or nine inches; and the seat of the stricture I have usually found to be about six or seven inches from the external meatus: occasionally I have found it anterior to this point, but rarely beyond it; and usually, where I have discovered stricture at a short distance from the anterior meatus, I have found a second at the distance of six or seven inches. The part of the urethra surrounded by the accelerator muscle is, I consider, more susceptible of disease than the other parts: it is abundantly furnished with mucous follicles, it is highly vascular, it is surrounded by a large mass of erectile tissue, which is again enclosed in the accelerator urinæ muscle; for this muscle does not merely lie upon the lateral and inferior portions of the bulb of the corpus spongiosum, but the lateral fibres are continued above the corpus spongiosum, where they are joined to a tendon which completes the circle. Besides, this portion of the urethra is extremely sensitive, so that the introduction of an extraneous body, as a bougie, whilst the urethra is in a healthy condition, usually produces pain in passing through this portion of the canal. The great sensibility of this part I consider to be designed for the purpose of exciting, by sympathetic influence, the action of the accelerator muscle during coition. We find the urethra in this situation to be more dilated than at any other part; and I conceive this to be for the reception of the fluids poured from the prostate, vesiculæ seminales, and vasa deferentia, which, accumulating in the part, excite spasmodic contraction of the muscle, which causes their forcible ejection. In accordance with this opinion, I think the old name of ejaculator seminis more applicable to the muscle than that now usually employed, of accelerator urinæ. In corroboration of my opinion, I would state that, in several instances in which I have known permanent stricture to exist, with an irritable condition of the urethra, patients have complained of severe pain in this part, at the time of the venereal orgasm during coition; and further, that in other patients, subject to permanent stricture in this part of considerable extent, the seminal fluids have not been ejected, although the orgasm has occurred, but have almost dribbled away subsequent to the orgasm.

*Treatment of Permanent Stricture.* In the first place, I must state that permanent stricture is very frequently supposed to exist when it does not, and that the means employed to relieve the supposed stricture frequently create a permanent disease. Very often have I seen young men who have supposed themselves to be the subjects of permanent stricture, from having read or heard accounts of the disease, and perhaps having been subject to gonorrhœa, which is so generally considered as the exciting cause. During the existence of gonorrhœa, either in the acute or chronic form, and even for a short time after the subsidence of all discharge, I grant that symptoms of permanent stricture may, and very frequently do exist; for, during the continuance of such disease, the portion of the mucous membrane from which the morbid secretion is poured out is somewhat thickened, and has its vascularity and sensibility increased; and even a trifling thickening of the membrane, in so small a canal as the urethra, must produce some alteration in the stream of urine; whilst, from sympathy of the bladder, there exists a more frequent desire to pass the urine. These symptoms also may exist when the discharge has subsided; for the membrane does not immediately recover its natural condition, nor does the sympathetic affection of the bladder immediately cease.

In my opinion, there are very few of these cases but would recover, unless for the interference of the surgeon. If we take a man in good health, and with sound urethra, and we introduce a bougie into the urethra with some little force, the progress of the bougie will be impeded, usually when it has passed to about the extent of six inches, or to that portion of the canal surrounded by the muscle; and, if force still be employed, the resistance becomes greater, and the membrane may be lacerated. The impediment arises from spasmodic contraction of the accelerator muscle, in consequence of the irritation of the mucous membrane by the bougie. If this will take place in the healthy urethra, (which I have ascertained by experiment,) how much more would it be likely to occur at a time when the membrane must be in a state of irritability. When therefore a patient is suffering from gonorrhœa or gleet, or is just recovered from such disease, but has slight symptoms of permanent stricture, if he unfortunately applies to a surgeon who is fond of the use of the bougie or sound, he is very likely to have some injury inflicted in the portion of the canal subject to spasm, which may give rise to permanent disease. One of my medical friends, during the period of his pupilage, after hearing a lecture on strictures, fancied himself to be the subject of the disease, and consulted a surgeon fond of the use of instruments. A moderate-sized bougie was introduced into his bladder without much difficulty; but the surgeon, not content with this, said that he must introduce a larger instrument before he could be satisfied. He accordingly endeavoured to pass a large sound; but, after repeated trials, and causing severe hemorrhage, he was obliged to desist. The patient had not previously been the subject of any urethral affection whatever, but has almost constantly been a sufferer since, during a period of more than twenty-five years.

I could relate many cases which have come under my own observation, in which instruments have been used when no stricture existed, and several in which the forcible use of the instrument has created formidable disease. Supposing the symptoms of permanent stricture to be decidedly developed, it is the duty of the surgeon to make an examination of the canal with the utmost caution and care; and the best instrument to effect this is the common wax bougie, which should be perfectly smooth and rendered moderately flexible by holding it in the hand for a short time; it should be smeared with sweet oil, and slightly curved towards the extremity which is to be introduced into the passage; in introducing it, (supposing the patient erect, and standing with his back against the wall of the apartment,) the penis should be drawn forwards, and somewhat upwards with the left hand, to put it on the stretch and bring it on the same plane as the perinæum. The point of the bougie being introduced into the urethra, it should be very gradually pushed on; the convexity of the curve (previously given to the instrument) being kept against the under part of the urethra, so that the point, as the bougie is carried on, passes along the superior part of the urethra. If any impediment arise, the surgeon should direct the point laterally or downwards, by slightly rotating the instrument between the finger and thumb, and, if he then find that he cannot pass it further, he should wait a few seconds, and then make a second attempt in the same way.

When the obstruction arises from permanent stricture, there is usually a sensation as if the point of the instrument struck against some hardened substance, and the bougie can be retracted with ease, but, when the obstruction is occasioned by spasm, the same sensation is not communicated to the surgeon, and the point of the instrument is grasped, so that it cannot be retracted without some force. Further, when the impediment proceeds from the existence of permanent stricture, the application of force is not productive of much suffering; whilst, in the case of spasm, the suffering is usually severe. In the first instance the impediment is not lessened by allowing the patient to rest; but, in the second, if gentle pressure be continued, the obstruction usually ceases.

It is best, in my opinion, to make the examination first with a bougie of moderate size, but if the first examination convinces the surgeon that permanent stricture exists, which prevents the passage of the instrument, he should select one of smaller size to enable him to pass the seat of stricture without violence. As the thickening which causes the stricture is most frequently partial, the course of the canal at the seat of disease generally deviates from the direct course, and it requires therefore a little manœuvring on the part of the surgeon to find its direction. It is not uncommon, when the contraction of the passage is considerable and irregular, for the surgeon to succeed in passing an instrument at one time with facility, and at another time to be altogether frustrated in his endeavour to do so. This may frequently be obviated by carefully observing the curve which the instrument has acquired in its introduction, and by giving a similar curve to any new instrument which may be employed.

When the first examination is made, if the surgeon finds the urethra to be exceedingly tender, and that hemorrhage occurs from the use of very slight force, he should not repeat his examination, but should adopt such local and general means as he may deem advisable to correct such condition of the part. I have generally found it in connexion with debility and morbid nervous excitement, requiring careful dietetic means, with the administration of tonics and narcotics generally, and local bloodletting by leeches, with evaporating lotions or fomentations. I am satisfied that no good can result from the continuance of mechanical interference under these circumstances, but that the mischief may be greatly augmented.

In the ordinary cases of permanent stricture, where the callous induration exists, many surgeons employ and recommend the use of metallic instruments in the outset of the treatment: such practice, even in the hands of the experienced surgeon, I consider to be attended with great risk; but, with the young and inexperienced surgeon, it is pregnant with the greatest danger. If the stricture be of very trifling extent, it may be sometimes rapidly subdued by such means; but, when it occupies a space of more than an eighth or a quarter of an inch, I am confident that more evil than good results from the practice. The diseased portion of the canal is, as I have observed, usually more resisting than the healthy part; and, if much force be employed, the healthy structure gives way, and the mischief thereby becomes extended. But this is not the only evil; for the urine may escape by the lacerated opening into the surrounding cellular tissue in small quantity, producing subsequent inflammation and suppuration, or to a great extent, so as to distend the cellular membrane of the perineum, scrotum, &c., and occasion sloughing, or such extensive disease as may be followed by great loss of structure; or, by creating constitutional disturbance, destroy life. I wish it to be distinctly understood that I am not here speaking theoretically but practically, having often witnessed the distressing and dreadful facts which I have described. My own practice is, cautiously to find my way through the stricture, at first with the common wax bougie, which I have made slightly conical at the extremity, to be passed into the urethra, and I persevere in the use of such instruments until I can pass one of moderate size with facility; when I resort to the use of the gum-elastic bougie, which offers more resistance, or to the sound. I prefer, however, the former. I do not consider either of them to be applicable to the early treatment of the disease.

I have not yet sufficiently tried the plan recommended by the Baron Dupuytren, of retaining a full-sized bougie in contact with the stricture for many hours together, to be able to speak confidently respecting it; but I am induced to believe that, when the stricture is not very extensive, it is likely to be serviceable; at all events, it cannot produce much mischief. I object to the use of caustic, or of the division of the stricture by a stilette, for the same reasons that I object to the sound; namely, the risk of perforating the wall of the canal, so as to allow of extravasation of urine.

